# Direct Hippocampal and Thalamic Inputs to Layer 3 Pyramidal Cells in the Medial Entorhinal Cortex Revealed by Monosynaptic Rabies Tracing

**DOI:** 10.1007/s12264-025-01363-x

**Published:** 2025-03-10

**Authors:** Ze Chen, Dietmar Schmitz, John J. Tukker

**Affiliations:** 1 Neuroscience Research Center, Charité-Universitätsmedizin Berlin, Corporate Member of Freie Universität Berlin and Humboldt-Universität zu Berlin, 10117 Berlin, Germany; 2 German Center for Neurodegenerative Diseases (DZNE) Berlin, 10117 Berlin, Germany; 3 Einstein Center for Neuroscience, Charité-Universitätsmedizin Berlin, Corporate Member of Freie Universität Berlin and Humboldt-Universität Berlin, 10117 Berlin, Germany; 4 NeuroCure Cluster of Excellence, Charité-Universitätsmedizin Berlin, Corporate Member of Freie Universität Berlin and Humboldt-Universität Berlin, 10117 Berlin, Germany; 5 Bernstein Center for Computational Neuroscience, Humboldt-Universität zu Berlin, 10115 Berlin, Germany

**Dear Editor**,

The importance of the medial entorhinal cortex (MEC) for memory and spatial navigation has been shown repeatedly in many species, including mice and humans [1, 2]. It is, therefore, not surprising that the connectivity of this structure has been studied extensively over the past century, mainly using a range of anterograde and retrograde anatomical tracers [3]. However, such approaches have limited resolution and cannot identify the inputs to specific cell types. More recently, patch-clamp recordings from pairs of MEC cells, as well as electron microscopy, are starting to reveal much of the local connectivity between MEC cell types [1]. Some non-local inputs onto specific MEC cell types have also been studied, revealing pathways for specific memory functions [2]. However, for most cell types in the MEC, we still do not have a whole-brain view of the inputs they receive. Particularly for the layer 3 (L3) pyramidal cells (L3Ps) that provide direct input to the hippocampal area CA1, which has been shown to be crucial for temporal association memory and other memory-related functions [2, 4], we know little about the non-local inputs beyond the neighboring presubiculum and parasubiculum [3]. Thus, we used monosynaptic rabies tracing [5, 6] in combination with whole-brain serial two-photon tomography (STPT) [7] to label the inputs to L3Ps from throughout the brain.

In Oxr1(oxidation resistance 1)-Cre mice, previously shown to express Cre in MEC L3Ps [4] (Fig. S1), we injected a Cre-dependent “helper” virus (*AAV1-Syn-FLEX-nGToG-WPRE3)* into the MEC, followed by a modified rabies virus ~3 weeks later (Fig. 1A). After another 8-11 days, we removed the brains and used STPT to reveal rabies-labelled cells in several brain areas (see Supplementary Information). These included the presubiculum (Fig. 1B), parasubiculum (Fig. 1C), and medial septum (Fig. 1D), all known to provide input to the superficial entorhinal cortex [1, 3]. More surprisingly, we also observed rabies-labelled presynaptic cells in hippocampal area CA1 (Fig. 1E), the subiculum (Fig.1F), and the anterodorsal nucleus (AD) of the thalamus (Fig. 1G), suggesting novel, direct monosynaptic input pathways from these areas to L3Ps in the MEC. Although the number of cells varied strongly between brains and sparse labelling was also occasionally seen in other brain areas, we found rabies-labelled cells in these brain areas in all analyzed hemispheres (*n =* 5 mice).

**Fig. 1:**
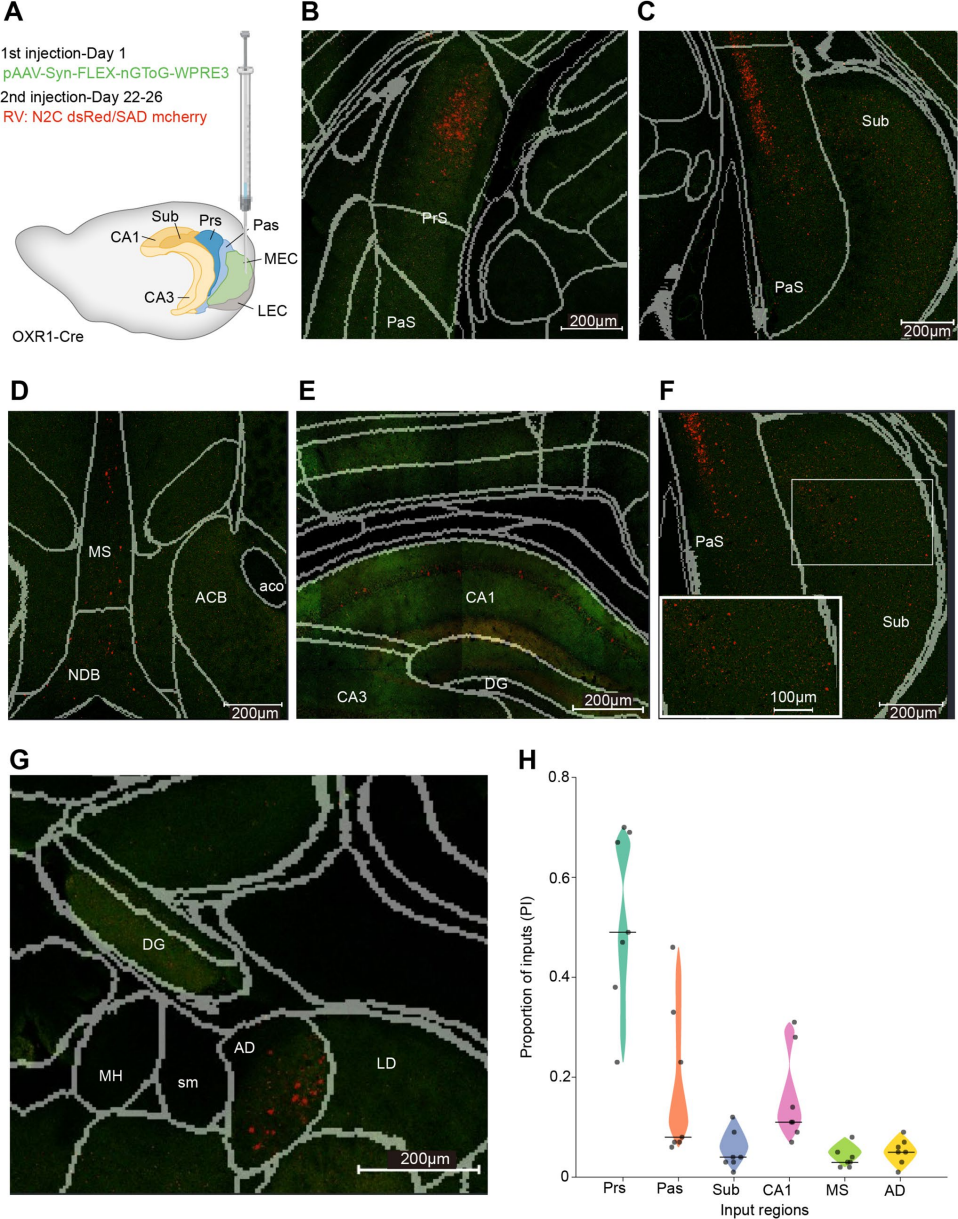
Global brain inputs to excitatory neurons in MEC L3 verified by Cre-dependent monosynaptic rabies virus (RV) tracing. **A** Schematic showing the injection strategy. The sagittal profile of the mouse brain showing the MEC in green together with neighboring structures: the lateral entorhinal cortex (LEC), presubiculum (PrS), parasubiculum (PaS), subiculum (Sub), and hippocampal areas CA1 and CA3. The MEC of an OXR1-Cre mouse is injected with pAAV-Syn-FLEX-nGToG-WPRE3 on Day 1, leading to nucleus-localized green fluorescence in infected cells at the injection site, followed by the rabies virus N2C dsRed/SAD mCherry on Day 22, leading to red fluorescence in rabies-infected cells throughout the brain. **B**–**G** Two-photon coronal images overlaid with registered boundaries (grey) from the Allen Brain Atlas, showing rabies-infected cells (red) in the PrS (**B**), PaS (**C**), medial septum (**D**) comprising the medial septum proper (MS) and the nucleus of the diagonal band (NDB), hippocampus (**E**), subiculum (**F**), and the anterodorsal nucleus (AD) of the thalamus (**G**). **H** Detected inputs from all 7 analyzed hemispheres, quantified as the proportion index (PI) following RV tracing in all brain regions with consistent labelling, except for the MEC. See Supplementary Table 1 for more information on the injection parameters.

We quantified these results by first registering the imaged 3D brain volumes to the Allen Mouse Brain Atlas using BrainReg (see Supplementary Information). Then, cells were counted manually. Due to the difficulty of differentiating true first-order (i.e., monosynaptic) presynaptic cells from second-order presynaptic cells and from starter cells with weak or no GFP labelling, we only counted the presynaptic cells located outside of the MEC injection site. Furthermore, since the absolute number of cells varied between brains, we summed all counted cells across the six regions and reported the proportion of cells in each region as the proportion index (PI) ranging from 0 to 1 (Fig. 1H; see also Fig. S2 for additional measures). Overall, the presubiculum provided the major input to MEC L3Ps: roughly half of the counted rabies-labelled cells were in the presubiculum (PI range 0.23–0.70, median 0.49). The parasubiculum provided only ~10% of the total input, although this number varied considerably (PI range 0.06–0.46, median 0.08). The medial septum also provided a minor but consistent input (PI range 0.02–0.08, median 0.04). Among the 3 novel input areas, cells from CA1 formed the greatest proportion (PI range 0.07–0.31, median 0.16), followed by the AD (PI range 0.01-0.09, median 0.05) and the subiculum (PI range 0.01–0.12, median 0.05).

For a more detailed analysis of the spatial distribution of labelled cells, we used a range of Brainglobe software tools (see Methods) and manual curation to plot the location of all cells in a standardized atlas space (Fig. 2). Overall, the distributions were surprisingly uniform across brains, particularly along the anteroposterior axis (panels iv and v in Fig. 2A–F; see also Fig. S3).

**Fig. 2:**
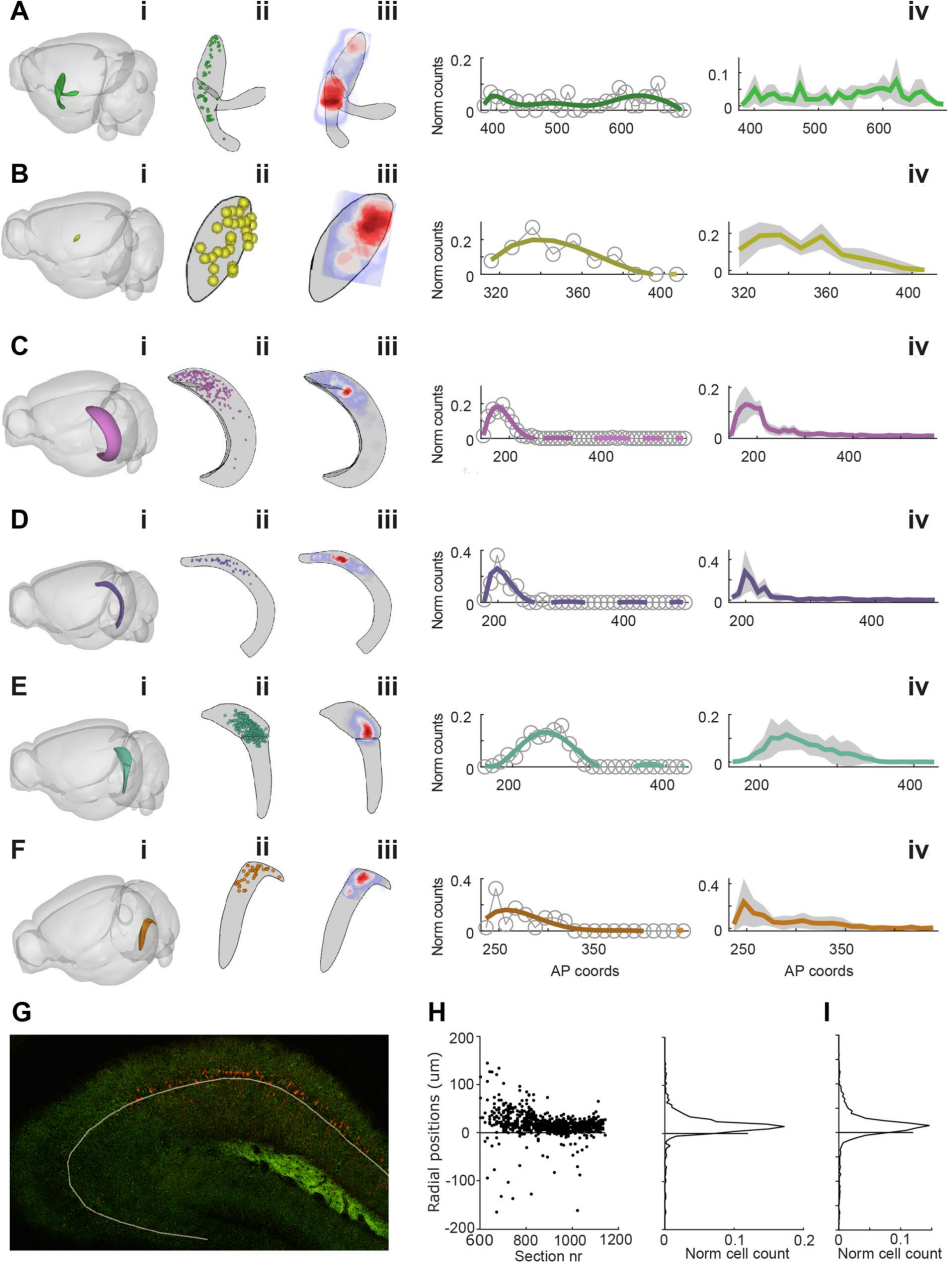
Spread of labelled neurons. For each of the six brain areas where we consistently observed rabies-labelled cells (**A–F**), we use Brainrender to plot the location of the area (**i**), the distribution of counted cells in this area from one example brain (**ii**), and the density of counted cells (**iii**). We also show the proportion of counted cells at different AP locations for the example cell (**iv**) and the mean distribution across all 7 analyzed hemispheres, with standard deviation. **G** Representative image showing the manually drawn line at the edge of stratum pyramidale and stratum radiatum used as reference for the radial plots in **D** and **E**. **H** Radial plot of the proportion of counted cells at different AP locations (left), with a histogram showing the overall distribution of cells as a function of radial distance. Zero indicates the stratum pyramidale/stratum radiatum border. **I** Overall distribution of cell counts as a function of radial distance for all analyzed hemispheres (*n =* 7) and cells (*n =* 1955).

In area CA1, the distribution was also heavily skewed, with a preference for the more anterior and dorsal parts of the hippocampus (Figs 2C and S4) The injection sites in the MEC extended across a much smaller extent of the anteroposterior axis (Fig. S5), although the boundaries of the injection area were somewhat imprecise, particularly since the GFP expression from the AAV starter cells could not always be reliably detected.

Along the radial axis of the hippocampus (i.e., orthogonal to the stratum pyramidale), the vast majority of neurons were located within the pyramidal cell layer (Fig. 2G–I), with no clear bias for a particular sublayer. The presence of cells almost exclusively in the stratum pyramidale suggests that the majority of rabies-labelled cells in CA1 consists of pyramidal cells. However, the stratum pyramidale is also known to contain GABAergic interneurons, which could, in principle, project extrahippocampally. Therefore, we applied immunohistochemical reactions with an antibody against GABA to sections from an additional rabies-injected Oxr1 mouse. We found that except for rare cells outside of stratum pyramidale, labelled cells did not express GABA (Fig. S6), strongly suggesting that they are indeed glutamatergic pyramidal cells.

We did not see labelled cells in the adjacent dentate gyrus (DG) or CA3. Since we could not reliably delineate the border of CA2 and CA1 without additional labeling, we use the term CA1 to indicate both CA1 and CA2. In general, some imprecision is inherent in the atlas registration process, and the locations of labelled cells in the standardized atlas are expected to deviate up to ~100 µm from their actual location [8]. Note that despite this limitation, we found a good overall match between region boundaries and clusters of labelled cells; even for the AD, a very small structure (Fig. 2B), we consistently found that the great majority of rabies-labelled cells in the thalamus fell within the registered borders (e.g., Fig. 1G).

The projection from CA1 to MEC L3Ps suggested by our rabies results was confirmed by additional AAV injections into CA1 of WT mice. We observed mScarlet expression in axons in the superficial MEC (Fig. S7), suggesting that, indeed, a subset of pyramidal cells project directly to the superficial MEC, likely making synaptic connections there with L3Ps.

In summary, our results suggest a limited but highly surprising set of inputs to MEC L3Ps, including not only inputs from the presubiculum, parasubiculum, and medial septum but also novel inputs from CA1, the subiculum, and the AD.

Inputs from the presubiculum to the MEC have been known for a long time [3, 9], and particularly, the previously described band of axons in L3 is consistent with the strong input we see onto L3Ps. Given the previously described band of axons from the parasubiculum in L2 and the fact that the apical dendrites of L3Ps extend from L3 down to L1, the generally much less abundant input from the parasubiculum was somewhat unexpected. The input from the medial septum is also consistent with several reports [1], although we are not aware of any direct demonstration of input from the medial septum specifically onto MEC L3Ps. In our case, the identity of the presynaptic cells could not be tested, but future work should aim to identify whether the medial septum cells are glutamatergic, GABAergic, or cholinergic since all three neurotransmitters have previously been shown to project to the MEC [1].

Although, as a proportion of total inputs, the input from AD was relatively modest, the density of cells providing input was quite high (Fig. 1G; see also Fig. S1D, E). This suggests that a large proportion of AD cells provide output to MEC L3Ps, in contrast to previous reports (e.g. [10]). If these cells have a high rate of divergence, even this relatively modest number of AD neurons may provide an important and previously undetected input to the MEC. AD is considered a core part of the so-called medial diencephalic-cortical memory stream underlying episodic memory [11]. The AD is also known to contain a large proportion of head-direction cells, which encode the direction an animal’s head is facing [12]. Clearly, input from the AD is likely to have important implications for the spatial coding and memory functions performed in the MEC, such as grid cells in MEC L3 [1].

Similarly, the inputs from CA1 and the subiculum to MEC L3Ps are also likely to impact spatial coding and memory function. Classically, inputs from these areas have been considered to be limited to the deep layers of the MEC, with only a few reports describing sparse axons extending more superficially in rats [3]. In contrast to the AD results, the density of labelling in CA1 was quite low (even more so in the subiculum; Fig. S2D, E), suggesting that only a specific subpopulation provides output to the MEC L3. What might this subpopulation be? While future studies are required to elucidate the functional properties of the hippocampal input cells, based on our data, we can say they include both CA1 and CA2 (but not CA3 or the DG; Fig. 1), are not restricted to any particular sublayer of the stratum pyramidale (Fig. 2G–I), and are mostly found in anterior CA1 (Figs 2C, S3, and S4). The latter observation suggests that the input may be spatial since the anterior dorsal (“septal”) pole of the hippocampus is known to contain more spatial coding cells than the ventral pole.

We therefore speculate that pyramidal cells in L3 of the MEC may receive both head-directional information from the AD and spatial information from CA1 (and CA2). In principle, such dual inputs could support the firing of grid cells, head direction cells, and conjunctive cells, all of which have been reported in L3 of the MEC (albeit mostly based on extracellular recordings with somewhat limited anatomical precision) [1]. It will be of particular interest to investigate the functional roles of the novel CA1 and AD inputs and their interplay *vis-à-vis* the previously described indirect inputs (e.g., from CA1 *via* MEC L5 or from the AD *via* the presubiculum) during memory and spatial navigation tasks.

Such investigation could apply novel rabies strains with lower toxicity to drive the expression of Ca^2+^ indicators or channelrhodopsins in presynaptic cells on the timescale needed for behavioral experiments [13, 14]. This would enable the identification of the presynaptic cell population coding and its effects on MEC coding. In a subset of injections, we have already confirmed that the novel rabies virus CVS-N2C strain [13, 14] shows results similar to the more classic SAD strain (see Supplemental Table 1). This not only paves the way for future experiments but also provides an extra control against potential tropism effects (see Supplementary Discussion for further methodological caveats).

We did not quantify local labelling in the MEC because of the difficulty of unambiguously identifying starter cells: it has been shown that even in the absence of fluorophore expression, the TVA receptor and glycoprotein expression can be sufficient for rabies tracing to work [15]. Furthermore, there is no way to distinguish “true” presynaptic cells if the rabies virus travels from the initial starter cell to another AAV-infected cell (as is likely to occur at the densely-labelled injection site (Figs S5B and S8C–E)); in such a case, the secondary AAV-infected cell will provide glycoprotein that will make the spread of rabies virus multisynaptic.

Overall, the robust presence of labelled cells in several previously-undescribed areas suggests that there are direct synaptic inputs from these areas onto MEC L3Ps, in contrast to the classical framework that has been textbook knowledge for decades. Such connectivity is likely to have important implications for the circuits underlying memory and navigation, which are involved in a host of diseases ranging from Alzheimer’s to epilepsy. At the very least, our work suggests novel targets that should be physiologically tested in future studies.

## Supplementary Information

Below is the link to the electronic supplementary material.

Supplementary file1 (PDF 1701 KB)

## Data Availability

The datasets generated during and/or analyzed during the current study are available from the corresponding author upon reasonable request.
