# Supplementary material for: Direct Hippocampal and Thalamic Inputs to Layer 3 Pyramidal Cells in the Medial Entorhinal Cortex Revealed by Monosynaptic Rabies Tracing
Source: Neurosci Bull. 2025 Mar 10;41(4):707–12. doi: 10.1007/s12264-025-01363-x (PMC11979043; doi:10.1007/s12264-025-01363-x)
Supplement: Supplementary file 1 — Supplementary file1 (PDF 1701 KB) [file 12264_2025_1363_MOESM1_ESM.pdf]

Supplementary Information

Materials and Methods

Oxr1-cre mice (C57BL/6N-Tg(Oxr1-cre)C14Stl/J; RRID:IMSR_JAX:030484; *n* = 5; 4 males, 1 female) were kept in their home cage with littermates, on an inverted 12:12 cycle with *ad libitum* access to food and water. To obtain the data for Fig. S1, an Oxr1-Cre mouse was cross-bred with a Rosa26$^{Lox\text{-}stop\text{-}LoxHTB}$ (R26) reporter mouse expressing histone-tagged green fluorescent protein (GFP) [16]; note that although these Oxr1-R26 mice also expressed the rabies glycoprotein B19G and the avian tumor virus receptor A (TVA) receptor, for this study we only reported the GFP expression profile as a readout of Cre-expression.

For viral injections (Supplementary Table 1), adult mice were deeply anesthetized with 2% isoflurane, and a craniotomy was made, exposing the transverse sinus at ~3 mm ML from the midline. An injection needle was slowly lowered 0.1-0.3 mm anterior to the edge of the sinus at an angle of 4° or 7° in the sagittal plane to a depth of 1.8 or 2.4 mm from the brain surface using a micromanipulator (Neurostar, Tübingen, Germany). Then we injected 100-200 nl of AAV (AAV1-Syn-FLEX-nGToG-WPRE3, BA-96; Charité Viral Core Facility, Berlin, Germany; https://vcf.charite.de/en/catalog/catalog_aav/aavs_ready_made/), to drive the expression of nuclear-localized GFP, TVA, and the mouse-optimized rabies glycoprotein [17,18]. We waited ~5 min before withdrawing the needle. Mice were provided with carprofen and metamizol and left to recover in their home cages. After 21-25 days we used the same procedure to inject 400 nl of Rabies (SAD-B19-dG-mCherry, BR-001 [5]; CVS-N2C-dG-dsRed [13], BR-24, RRID:Addgene_73460; Charité Viral Core Facility). We used two methods for viral injection: Nanofil syringes with 34G beveled needles (WPI, USA), at a rate of 40-50 nl/min (Neurostar), or pulsed pressure injection using a Picospritzer III system (Parker Hannifin, Hollis, NH, USA; RRID:SCR_018152) and a glass pipette (tip inner diameter, 20–30 μm) at a rate of 20-30 nl/min with a 10-ms pulse duration [19].

Another set of injections was performed in adult male C57BL/6N mice (*n* = 2), using the same procedures to inject 100 nl of AAV9-syn-OPN3(WT)-ER-mScarlet-Ts-WPRE (654c; Charité Viral

Core Facility, Berlin, Germany) into CA1 (in mm relative to Bregma: AP, 2.2; ML, 1.4; DV, 1.4). After 29 days the mice were perfused, then horizontal sections were cut at 70 µm on a vibratome (VT1200 S, Leica Biosystems; RRID:SCR_018453), mounted on glass slides with DAPI-containing mounting medium, and imaged under an epifluorescence microscope (Leica Thunder; RRID:SCR_023794).

In one additional case, we injected a male adult Oxr1-Cre mouse as above, using a glass pipette to inject bilaterally <100 nl AAV (AAV9-Syn-FLEX-nGT.N2cG-WPRE3 (BA-334; Charité Viral Core Facility) into the MEC, and 34 days later 200 nl of the CVS-N2C rabies BR-24 was injected through a needle into the left MEC. The mouse was perfused 15 days after the rabies injection, the brain was cut coronally at 70 µm, and one day later sections were washed in PBS, incubated in a blocking solution (5% normal goat serum (Biozol), 1% Triton-X (Sigma-Aldrich), and PBS) for 3 h at room temperature (RT) with gentle agitation, and incubated for 24 h at RT in a solution containing a primary polyclonal guinea pig antibody against GABA (ab17413, Abcam; RRID:AB_443865) diluted 1:300 or 1:400 in blocking solution (2.5% normal goat serum, 1% Triton-X, PBS). Next, the sections were washed again in PBS before incubation for 3 h at RT in a solution containing secondary polyclonal goat anti-guinea pig AlexaFluor-647 antibody (A-21450, ThermoFisher; RRID:AB_2535867) diluted 1:500 in PBS. Finally, the sections were washed 4 times with PBS (15 min), before being mounted on glass slides in mounting medium (Mowiol) and imaged under an epifluorescence microscope as above.

Mice were perfused 8-11 days after the rabies injection. They were first given an overdose of ketamine/xylazine and then perfused transcardially with 0.1 M PBS followed by 4% paraformaldehyde (PFA). Brains were kept in PFA overnight, then cut coronally at 40 µm and imaged 8 times per section (5 µm per optical section, pixel size 2 µm × 2 µm) using a custom-made STPT setup[7], at 920 nm (Ti:Sapphire Chameleon Ultra laser, Coherent, Dieburg, Germany), controlled *via* BakingTray (https://github.com/SWC-Advanced-Microscopy/BakingTray) and ScanImage (Vidrio Technologies, USA; RRID:SCR_014307). Images were stitched together using StitchIt (https://github.com/SWC-Advanced-Microscopy/StitchIt) and viewed using Napari (https://github.com/napari/napari)[20].

The full-brain 3D image set was then registered to the Allen Mouse Brain Common Coordinate Framework (CCFv3; RRID:SCR_020999)[21] or the Kim mouse brain atlas [22] which is based on the Paxinos and Franklin Mouse Brain atlas [23] using Brainreg https://github.com/brainglobe/brainreg[8], which is based on aMAP[24] and makes use of the BrainGlobe API (RRID:SCR_023848)[25]. Figures were created with Adobe Illustrator (RRID:SCR_010279), Inkscape (RRID:SCR_014479), Biorender (RRID:SCR_018361) and chiplot.online**.**

Virally-labelled presynaptic cells in different brain regions were manually counted using the cell-counter tool (https://imagej.net/plugins/cell-counter) in Fiji[26] (RRID:SCR_003070) in every 20th registered optical section (100 µm). We calculated the proportion index (PI) as the ratio of the number of labeled presynaptic neurons in a brain region of interest *versus* the overall total labeled neurons in each case. A second method of counting made use of Brainglobe tools (https://brainglobe.info/) to enable more detailed anatomical analyses. For this, we first ran Cellfinder[8,27] for automated atlas registration and detection of possible cells, then manually selected correctly classified cells using the curation tool of the Napari Cellfinder plugin, and finally the Brainmapper cell transformation widget in Napari to map the detected cell locations to the standardized atlas space. Finally, we plotted detected cells, cell density, and selected brain structures using Brainrender[28] and applied additional analyses with custom-written Python and MatLab (The Mathworks Inc; SCR_001622) scripts. To analyze the radial location of labelled cells in CA1, we created a new Napari shapes layer and manually drew a path tracing the border of the stratum pyramidale and stratum radiatum in the background GFP channel. A custom-written script was used to calculate the shortest (i.e. radial) distance between this line and all cells in CA1-2.

Supplementary Discussion: Methodological Caveats

In general, tropism effects have been described for most viruses, and rabies is no exception[14,29,30]. Thus, the exact proportions we report for inputs from different areas could theoretically be influenced by such effects. However, a large amount of data suggests that the rabies method effectively labels monosynaptic connections[31], so that at least qualitatively the novel connections we report are unlikely to be artefactual. Other confounding factors could be the precise location of our injection site, Cre

expression in the Oxr1-Cre line outside of MEC L3, or non-specific infection by the AAV helper virus due to limitations of the Cre-FLEX interaction. Regarding the latter issue, we performed a series of control experiments with different AAV dilutions (since leaky expression is more likely at higher titers), as well as with a Tet-TRE dual virus approach reported to address this issue[15], leading us to conclude that expression of our AAV helper virus was limited to Cre-expressing neurons (data not shown). Regarding the injection locations in the MEC, injection sites could indeed extend anteriorly to include a few cells in the subiculum or along the needle track in the visual cortex, and we did see Cre expression in the subicular complex after crossing our Oxr1-Cre line with a reporter mouse (Fig. S1). The fact that we occasionally saw sparse rabies-labelled cells in other brain areas such as the claustrum, suggests that in some cases a small number of starter cells was located outside of MEC L3 (Fig. S8). However, these "off-target" effects were inconsistent across samples, depending on random variations that were only seen in some cases (e.g. claustrum labelling when we had starter cells in the visual cortex). In contrast, all areas we report here were seen consistently across all analyzed samples (e.g. CA1 labelling was present regardless of whether starter cells were present in the subiculum). Thus, although we cannot exclude a minor effect from off-target starter cells, overall our findings cannot be explained by off-target effects. On the other hand, the variation in the PI measurement between individuals may be explained by more subtle variability in targeting, both of the rabies and the helper AAV injections (Figs 1H and S2). Different batches of AAV or rabies strains, with different titers, may also have contributed to the variability, although we did not systematically investigate this. Finally, although both genetically and in terms of experience the mice in our study were quite similar, it cannot be excluded that some of the variability we observed reflects true biological variability. It will be interesting to see in future studies to what extent and under which circumstances the connectivity may change.

Supplementary Figure Legends:

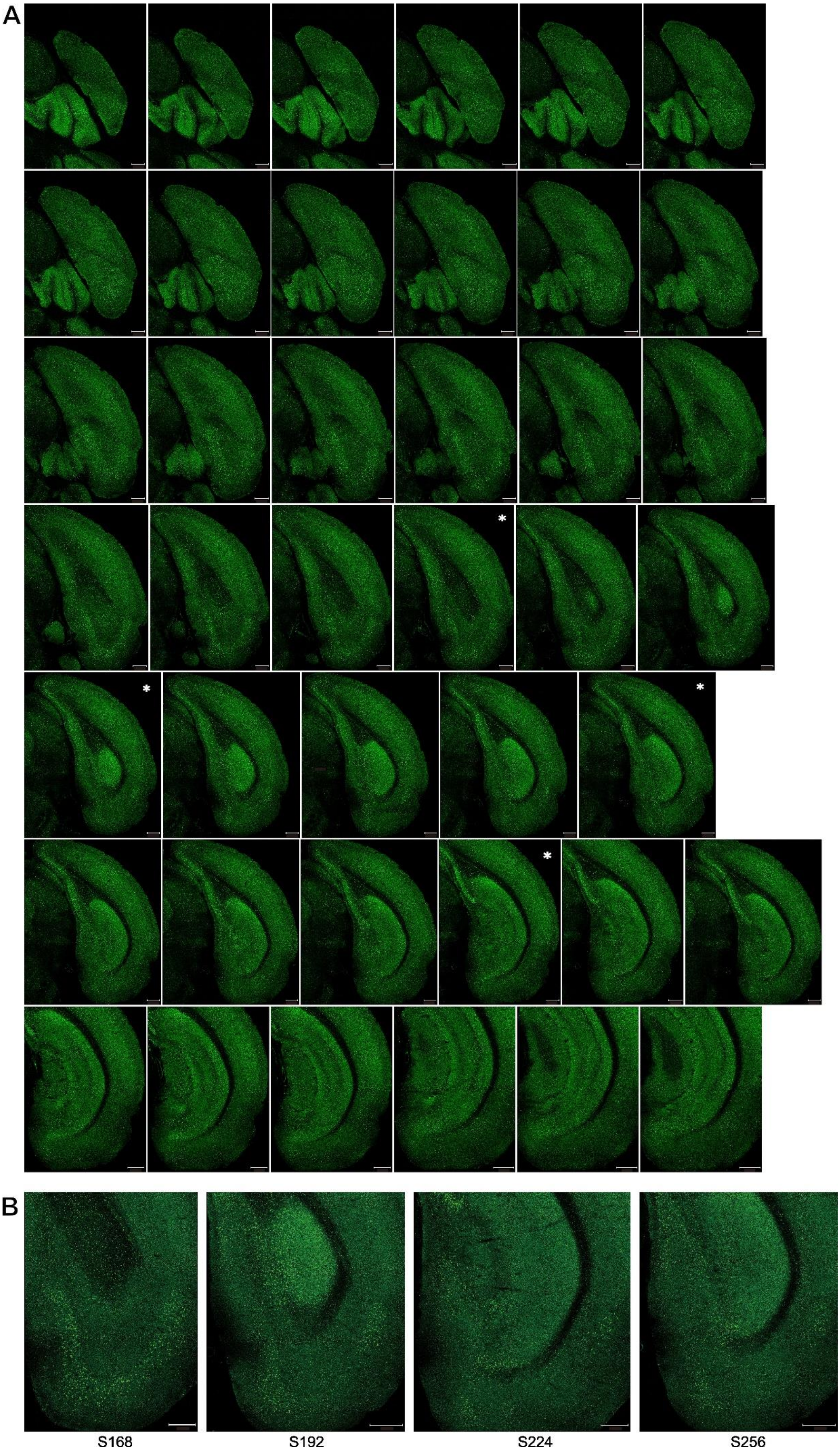
A
B
S168
S192
S224
S256

Fig. S1 Representative expression profile in Oxr1-R26 mice, showing soma-localized GFP (green) in Oxr1-Cre cells, consistent with previously published work[4]. **A** Coronal sections are ordered from posterior (upper left) to anterior (lower right). Every 8th optical section is shown. Note in the more posterior sections clear expression in the ventral part, comprising the MEC, but not in the dorsal part, comprising the visual cortex. Note also selective labelling in L3. In the more anterior sections, labelling also occurs in subicular complex areas. Asterisks indicate sections shown at higher magnification in B. **B** Close-ups of selected sections in **A**. Image S168: clear somatic GFP labelling in MEC L3. Note that weaker non-selective background labelling can be clearly distinguished from true somatic GFP expression. Images S192, S224, S256: clear somatic GFP labelling in MEC L3, but also in the presubiculum, subiculum, and subicular transition area. Scale bars A, Bii, iv, 200 µm; Bi, iii, 150 µm.

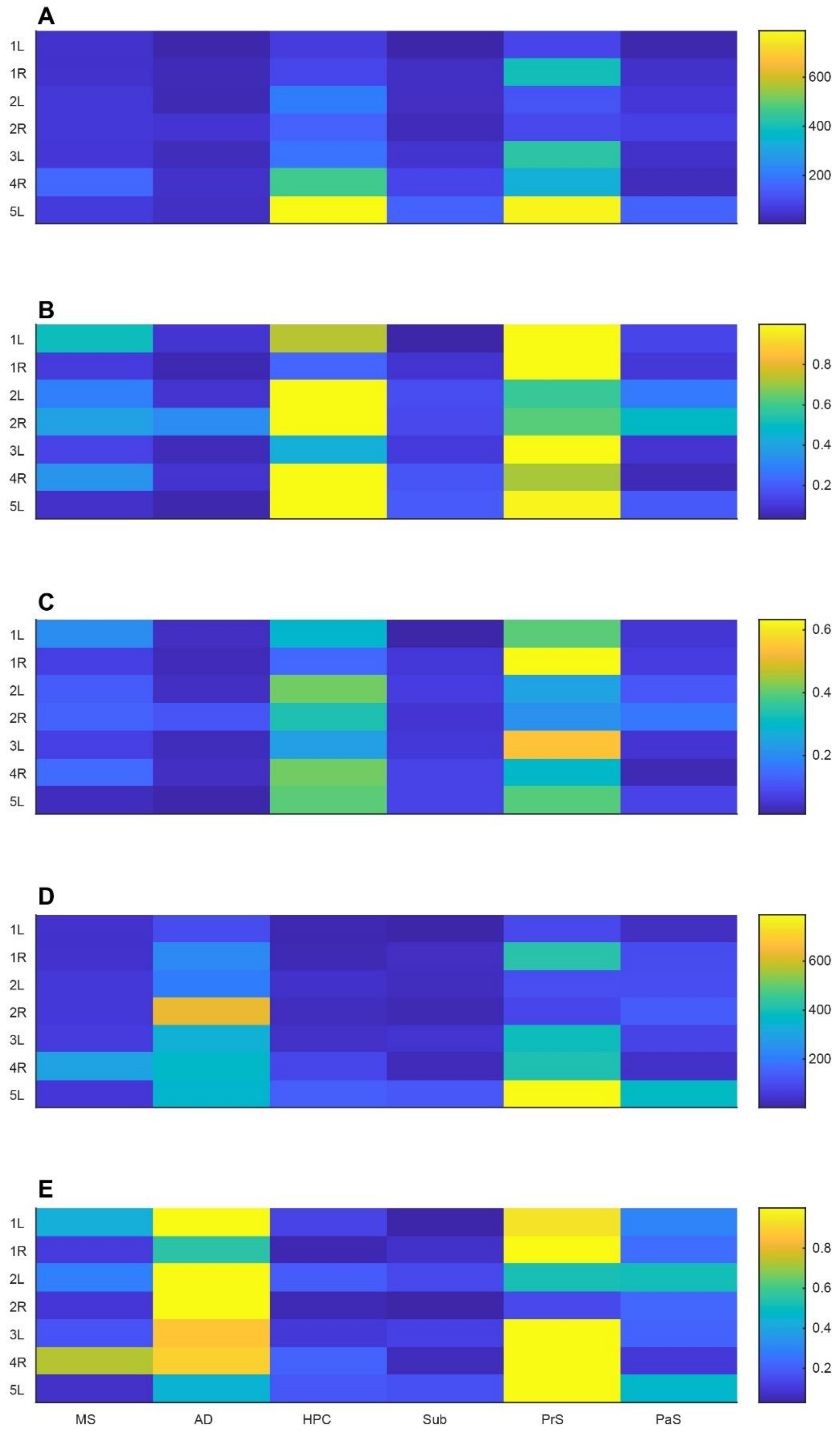
A
B
C
D
E
1L
1R
2L
2R
3L
4R
5L
MS
AD
HPC
Sub
PrS
PaS
200
400
600
0.2
0.4
0.6
0.8

Fig. S2 Numbers of presynaptic cells in 6 brain areas across all 7 hemispheres, with various normalizations. **A** Raw cell counts. **B** Cell counts divided by the total counts across all areas per hemisphere; referred to as the proportion index PI, this is the main measure we use in the rest of the manuscript. **C** Relative cell counts, i.e. cell count per brain area divided by the highest count across all areas per hemisphere. **D** Cell counts divided by the volume of each brain area, i.e. the density of counted cells per area. **E** Relative density, i.e. density per brain area divided by the highest density across all areas per hemisphere. MS, medial septum; AD, anterodorsal nucleus of the thalamus; HPC, hippocampus; Sub, subiculum; PrS, presubiculum; PaS, parasubiculum.

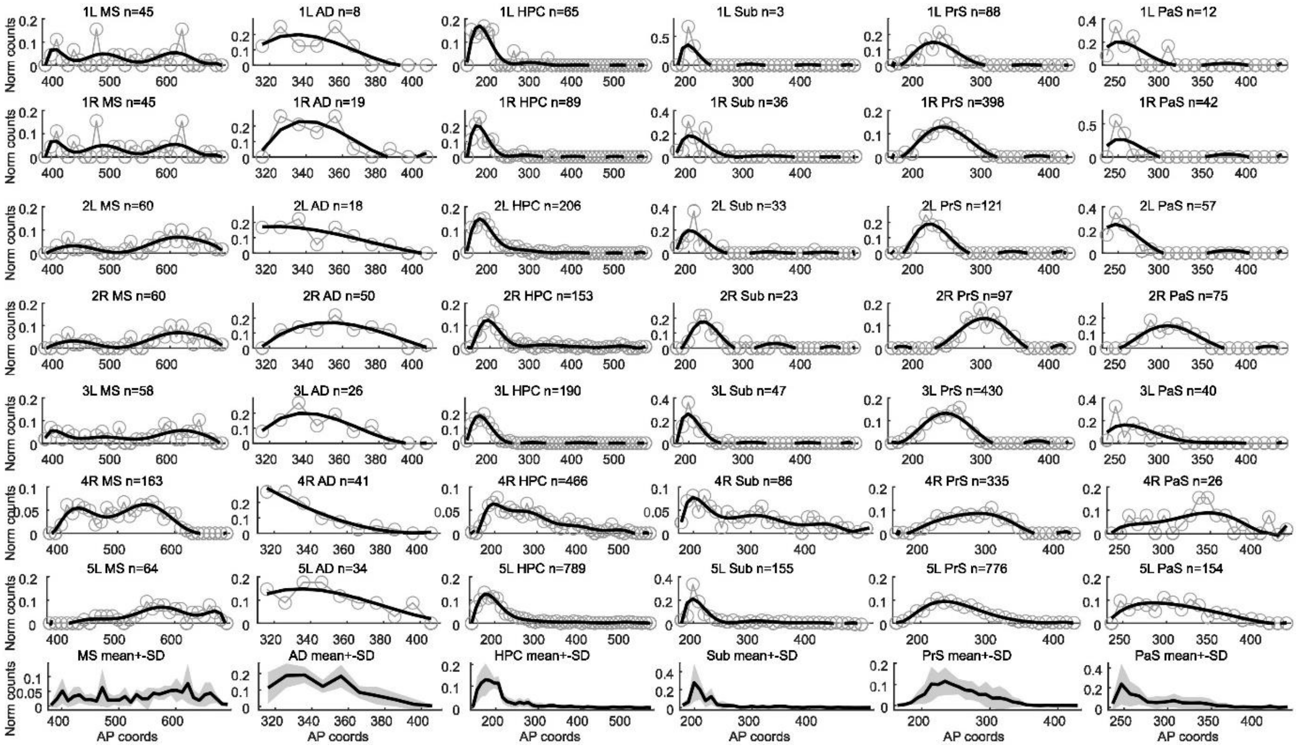

Fig. S3 Numbers of presynaptic cells as a function of anteroposterior (AP) location in standardized atlas space, plotted for all brain areas and hemispheres (delineated as 1L, 1R, etc.). Note that the distributions are relatively consistent across all hemispheres. In all plots except the bottom row, thick black lines are polynomials, generated with the MatLab polyfit function with degree nbins/4; circles mark the normalized cell count for each counted section (i.e. cell count per section divided by the total cell count per brain area); "*n*" refers to the total cell count per brain area. In the lower row plots, the mean normalized cell count over all seven hemispheres is shown as a black line, with the standard

deviation (SD) shown in grey. MS, medial septum; AD, anterodorsal nucleus of the thalamus; HPC, hippocampus; Sub, subiculum; PrS, presubiculum; PaS, parasubiculum.

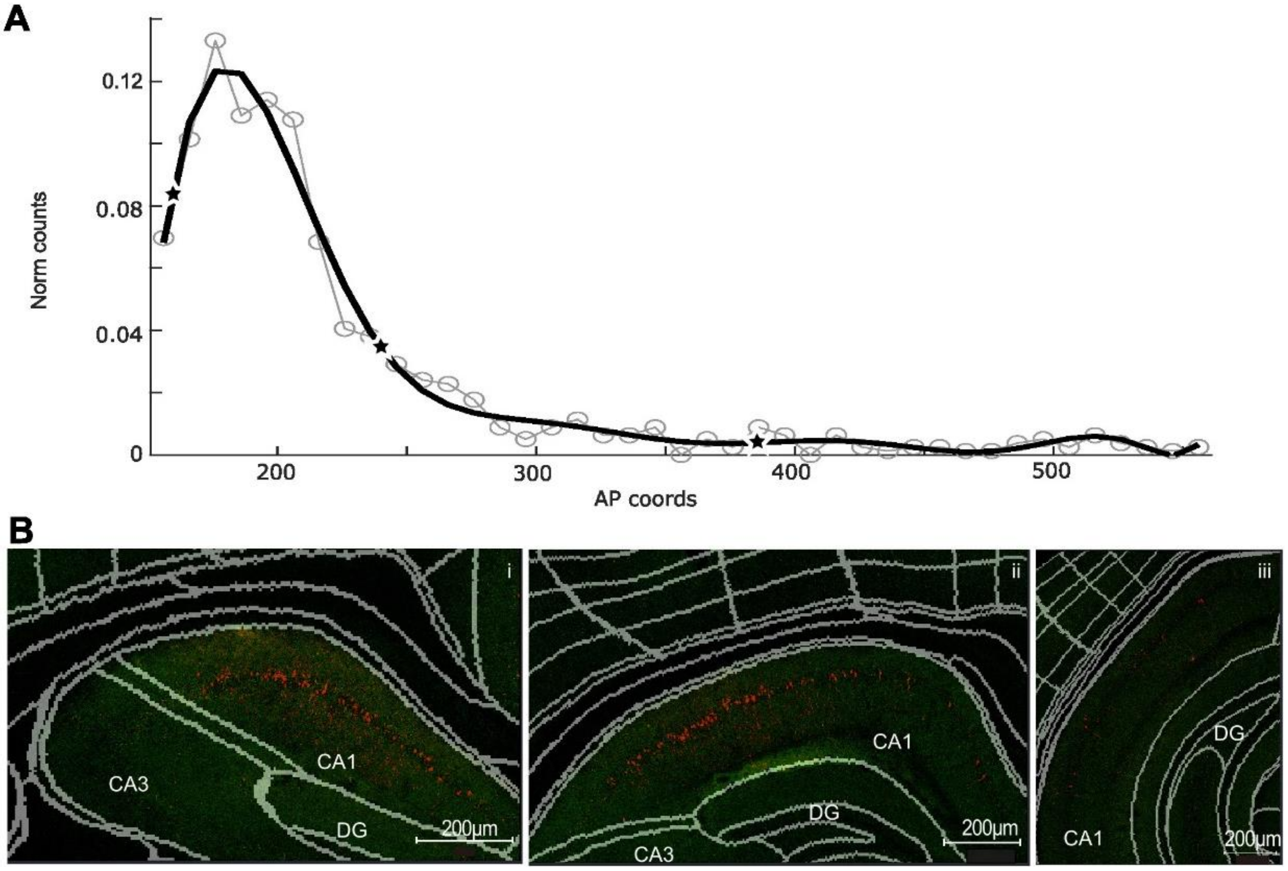

Fig. S4 Representative rabies-labelled presynaptic neurons in CA1 at different anteroposterior (AP) locations. **A** Normalized cell counts from a single hemisphere. Asterisks mark the optical sections from which the images shown in B were taken. **B** CA1 presynaptic neurons (red) labeled at 3 sites along the AP axis ranging from anterior dorsal CA1 (i) to posterior intermediate CA1 (iii). CA1, cornu ammonis 1; CA3, cornu ammonis 3; DG, dentate gyrus.

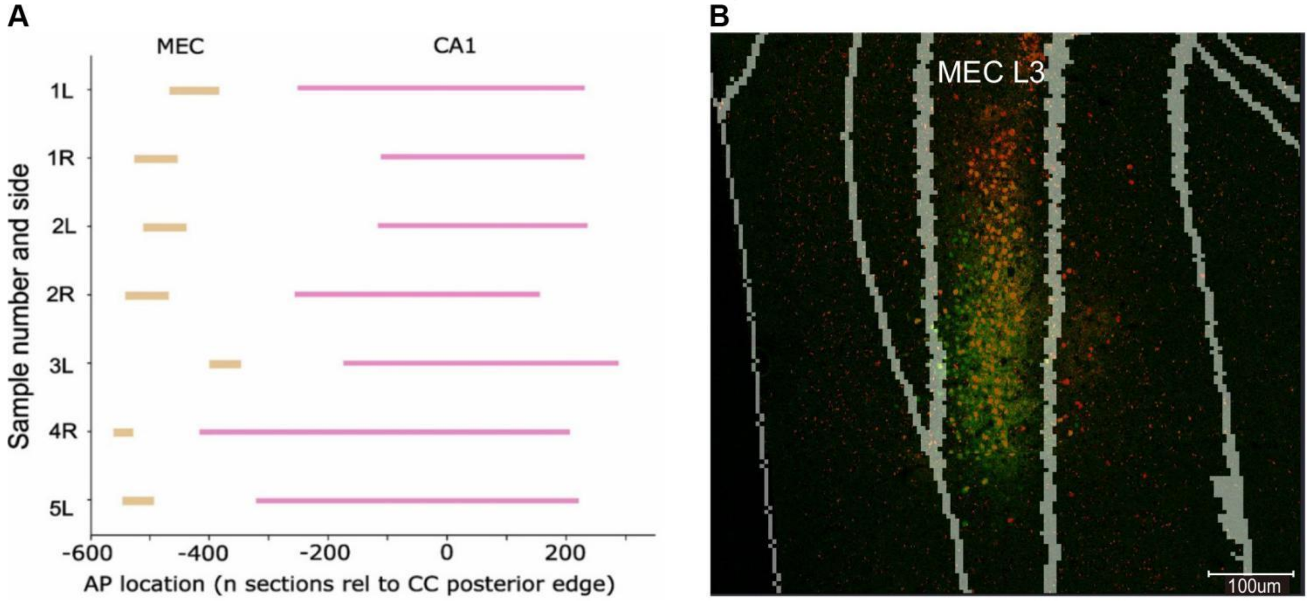

Fig. S5 Injection site in the medial entorhinal cortex (MEC). **A** All optical sections (thickness 5 μm) for which we detected presynaptic neurons in CA1, together with optical sections marking the injection sites in the MEC, for all 7 hemispheres, as a function of anteroposterior (AP) location. Coronal sections are numbered relative to the posterior edge of the corpus callosum (CC), taken here as an anatomical reference point. **B** AAV-infected non-rabies-labelled cells (green), presynaptic rabies-labelled cells (red), and starter cells (yellow) at the injection site in MEC L3. Note that the starter cells are mostly limited to MEC L3, as delineated by Brainreg (see Supplementary Information). CA1, cornu ammonis 1; MEC L3, layer 3 of the medial entorhinal cortex.

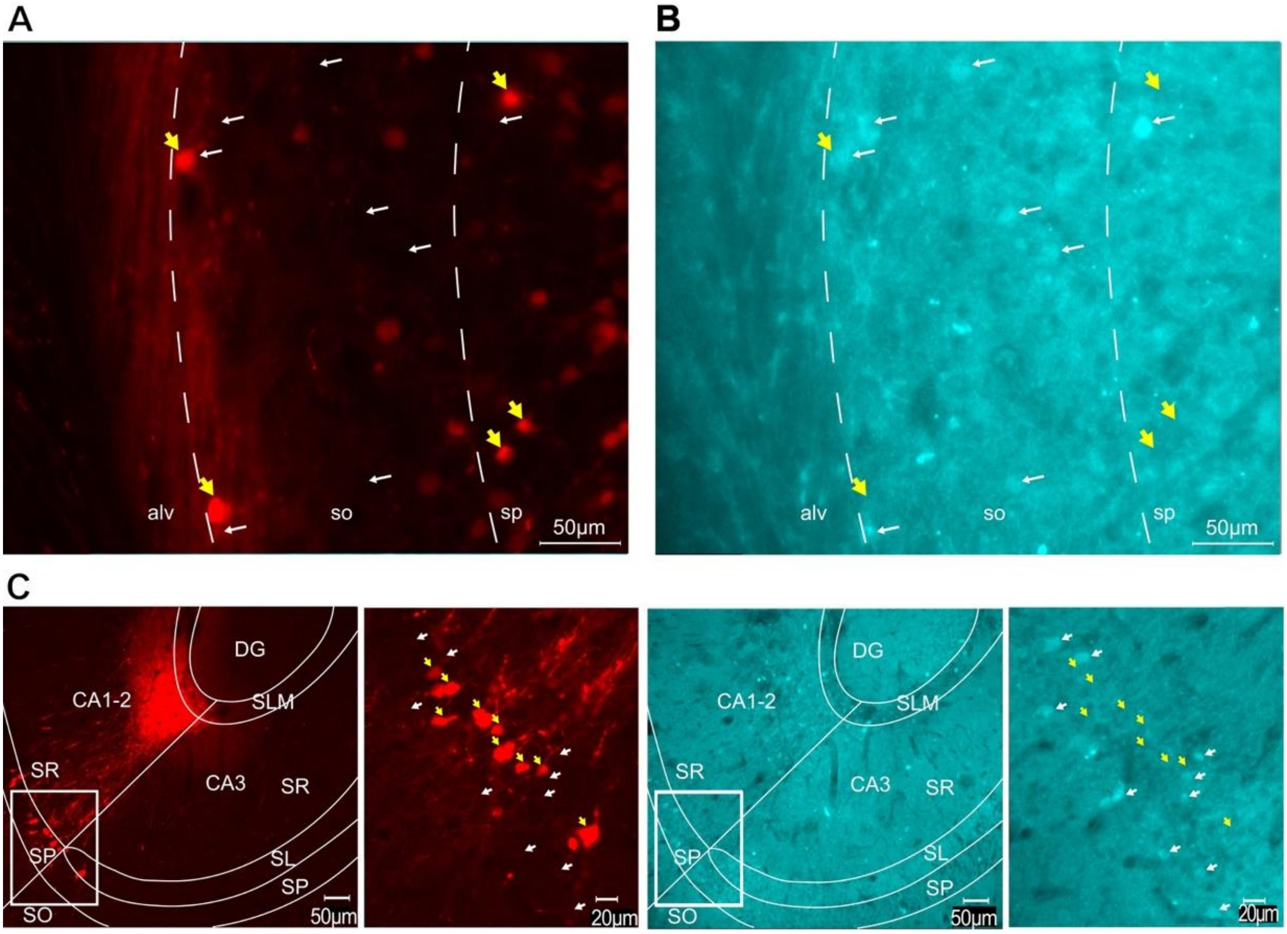

Fig. S6 GABA-immunolabelling of rabies-labelled cells. **A** Immunohistochemical results showing rabies-labelled cells (red, yellow arrows) and the locations of GABA+ cells (white arrows) in CA1-2. Note the lack of overlap, except for one cell in the stratum oriens (SO). **B** GABA immuno-labelling (cyan, white arrows) for the same field of view as in A. Note that the GABA immuno-reaction likely does not label all GABAergic cells, and sometimes GABA-immunopositivity could not be confirmed (bottom neuron in the SO, near the alveus (alv)) even though it is very unlikely that cells outside of the stratum pyramidale (sp) do not express GABA. For some out-of-focus cells in the sp (yellow arrows), a slight reduction in background staining is likely to indicate GABA-immunonegativity. **C** Further examples of rabies-labelled (yellow arrows) and GABA+ cells (white arrows) in CA1-2. Two left images show rabies-labelling (red), with the second image showing the rectangular area in the first image at higher magnification. The same applies to the two right images, which show GABA immuno-labelling (cyan). Note the lack of overlap. SP, stratum pyramidale; SR, stratum radiatum; SLM, stratum lacunosum moleculare; SL, stratum lucidum; CA, cornu ammonis; DG, dentate gyrus.

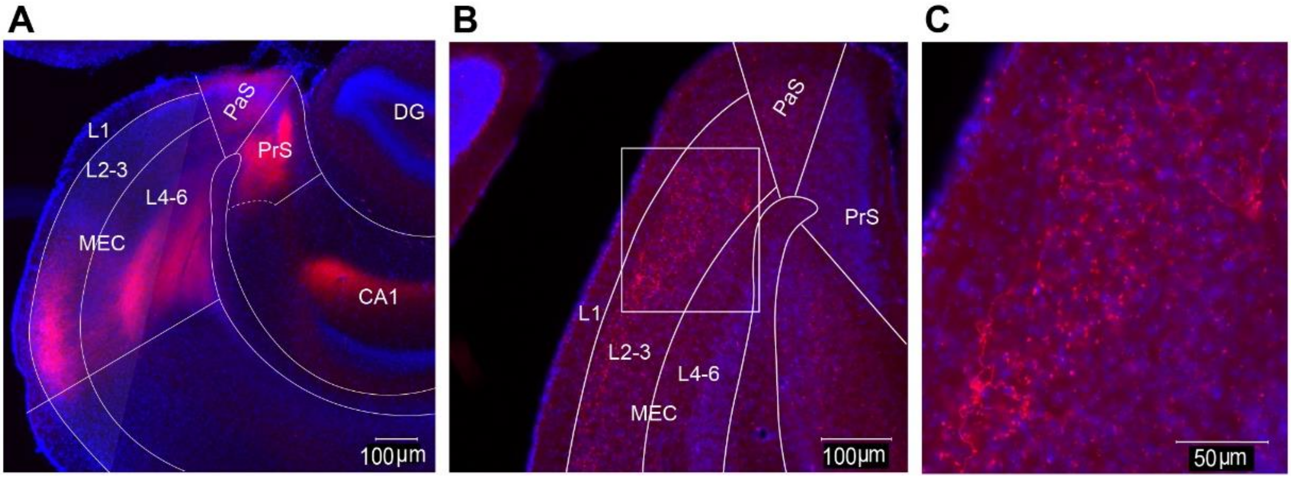

Fig. S7 Axonal labeling driven by AAV injections into CA1 reveal projections to the superficial MEC. **A** Representative horizontal section showing axonal labelling (red) in the superficial MEC. Layers and area borders were estimated based on DAPI staining (blue). Note labelling is also seen in the deep layers of the MEC, as previously described. **B** Example from a second injected mouse also showing axonal labeling in the superficial MEC. White box marks the area shown magnified in panel C. **C** Single axons in the superficial layers of the MEC. MEC, medial entorhinal cortex; L, layer; PaS, parasubiculum; PrS, presubiculum; CA, cornu ammonis; DG, dentate gyrus.

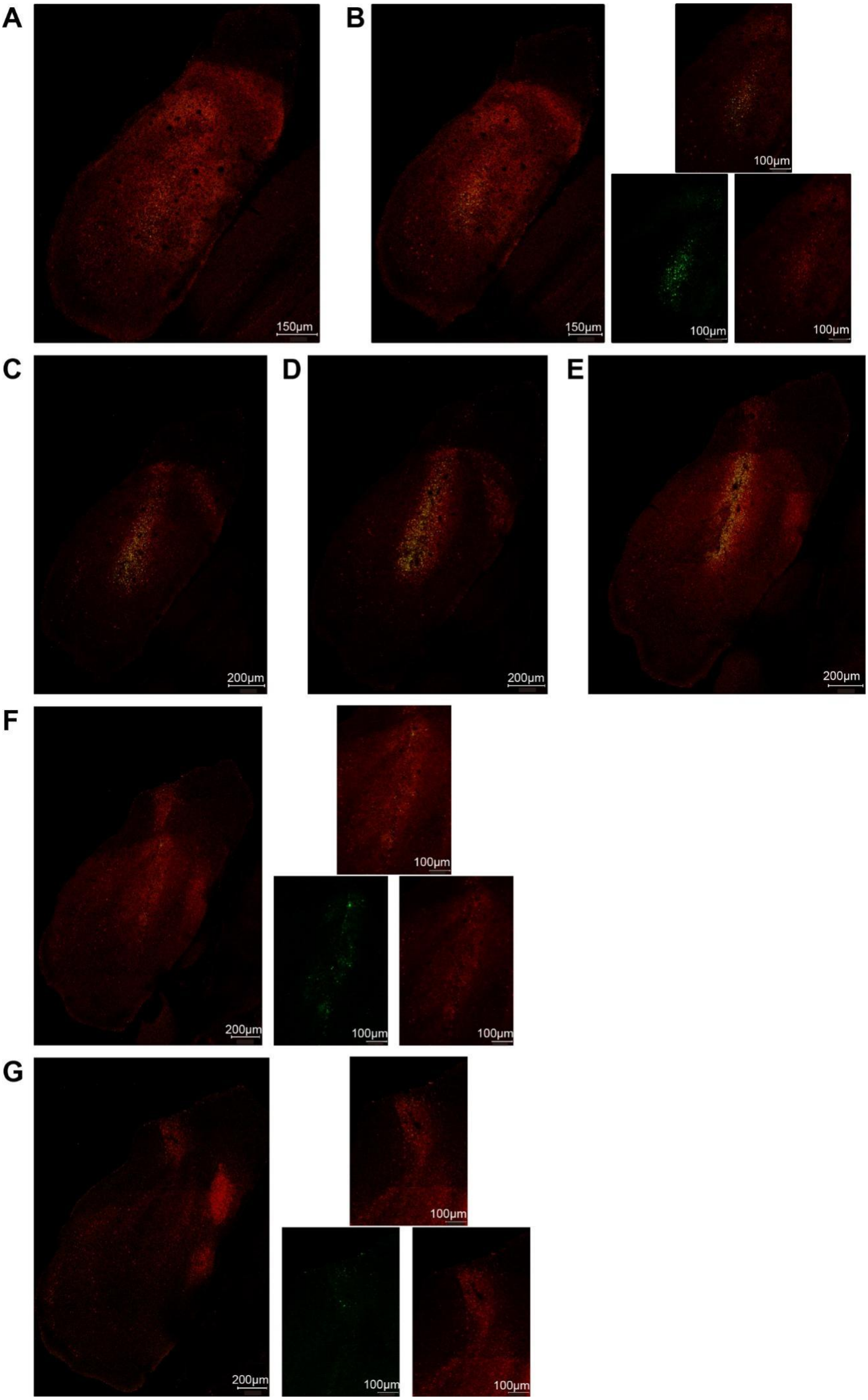

A
150µm
B
150µm
100µm
100µm
100µm
C
200µm
D
200µm
E
200µm
F
200µm
100µm
100µm
100µm
G
200µm
100µm
100µm
100µm

Fig. S8 Example MEC injection site (sample 5L), showing the somatic localization of GFP (green) in AAV-infected Oxr1-Cre cells and dsRed (red) in rabies-infected cells. Starter cells expressing both fluorophores (yellow) are the presumed postsynaptic sites for all ds-Red labelled cells throughout the brain. Optical coronal sections are ordered from posterior (**A**, upper left) to anterior (**G**, lower). **A** Most posterior optical section for which we could detect starter cells. **B** Optical section with a larger number of starter cells (yellow), shown magnified on the right (lower left, GFP expressing Oxr1-Cre cells; lower right, dsRed-expressing Rabies cells; upper, overlay). **C-E** Optical sections of a further injection site (note every 16th section is shown). **F** Most anterior optical section showing substantial starter cell labelling. **G** Representative optical section anterior to F, showing no starter cells except for very sparse labelling along the injection needle track.

Table S1: Virus Injection Parameters

Injected mouse # and hemisphere	1L	1R	2L	2R	3L	4R	5L
AAV volume (nl)	200	200	200	200	200	100/100	50/50
Days after AAV injection	25	25	25	25	25	21	21
RV volume (nl)	400	400	400	400	400	400	400
RV type	SAD-mCherry	SAD-mCherry	SAD-mCherry	SAD-mCherry	SAD-mCherry	N2C-dsRed	N2C-dsRed
Days after RV injection	10	10	10	10	10	8	11
Injection method	Needle	Needle	Needle	Needle	Needle	Glass pipette	Glass pipette
Injection angle	4°	4°	4°	4°	4°	7°	7°
Injection coordinates:							
AP (mm anterior to TS)	0.1	0.1	0.1	0.1	0.1	0.2	0.3
ML (mm posterior to Bregma)	3.3	3.3	3.3	3.3	3.3	3.1	3.1
DV (mm from dura)	1.8	1.8	1.8	1.8	1.8	1.4 / 2.4	1.4 / 2.4

Mice are identified by numbers 1-5, with L and R indicating left and right hemispheres, respectively. Further abbreviations: AAV, adeno-associated virus; RV, rabies virus; AP, anteroposterior; TS, transverse sinus; ML, mediolateral; DV, dorsoventral.

References

16. Li Y, Stam FJ, Aimone JB, Goulding M, Callaway EM, Gage FH. Molecular layer perforant path-associated cells contribute to feed-forward inhibition in the adult dentate gyrus. Proc Natl Acad Sci USA 2013, 110: 9106–9111.
17. Kim EJ, Jacobs MW, Ito-Cole T, Callaway EM. Improved monosynaptic neural circuit tracing using engineered rabies virus glycoproteins. Cell Rep 2016, 15: 692–699.
18. Zolnik TA, Ledderose J, Toumazou M, Trimbuch T, Oram T, Rosenmund C, *et al*. Layer 6b is driven by intracortical long-range projection neurons. Cell Rep 2020, 30: 3492–3505.e5.
19. Lin X, Cyrus N, Avila B, Holmes TC, Xu X. Hippocampal CA3 inhibitory neurons receive extensive noncanonical synaptic inputs from CA1 and subicular complex. J Comp Neurol 2023, 531: 1333–1347.
20. Sofroniew N, Lambert T, Evans K, Nunez-Iglesias J, Bokota G, Winston P, *et al*. napari: a multi-dimensional image viewer for Python. Zenodo 2022.
21. Wang Q, Ding SL, Li Y, Royall J, Feng D, Lesnar P, *et al*. The Allen mouse brain common coordinate framework: A 3D reference atlas. Cell 2020, 181: 936–953.e20.
22. Chon U, Vanselow DJ, Cheng KC, Kim Y. Enhanced and unified anatomical labeling for a common mouse brain atlas. Nat Commun 2019, 10: 5067.
23. Paxinos G, Franklin KBJ. Paxinos and Franklin's the mouse brain in stereotaxic coordinates. 4th ed. Amsterdam: Boston, 2013.
24. Niedworok CJ, Brown APY, Jorge Cardoso M, Osten P, Ourselin S, Modat M, *et al*. aMAP is a validated pipeline for registration and segmentation of high-resolution mouse brain data. Nat Commun 2016, 7: 11879.
25. Claudi F, Petrucco L, Tyson A, Branco T, Margrie T, Portugues R. BrainGlobe Atlas API: A common interface for neuroanatomical atlases. J Open Source Softw 2020, 5: 2668.

26. Schindelin J, Arganda-Carreras I, Frise E, Kaynig V, Longair M, Pietzsch T, *et al*. Fiji: An open-source platform for biological-image analysis. Nat Methods 2012, 9: 676–682.

27. Tyson AL, Vélez-Fort M, Rousseau CV, Cossell L, Tsitoura C, Lenzi SC, *et al*. Accurate determination of marker location within whole-brain microscopy images. Sci Rep 2022, 12: 867.

28. Claudi F, Tyson AL, Petrucco L, Margrie TW, Portugues R, Branco T. Visualizing anatomically registered data with brainrender. Elife 2021, 10: e65751.

29. Qiu L, Zhang B, Gao Z. Lighting up neural circuits by viral tracing. Neurosci Bull 2022, 38: 1383–1396.

30. Liu Q, Wu Y, Wang H, Jia F, Xu F. Viral tools for neural circuit tracing. Neurosci Bull 2022, 38: 1508–1518.

31. Callaway EM, Luo L. Monosynaptic circuit tracing with glycoprotein-deleted rabies viruses. J Neurosci 2015, 35: 8979–8985.
